# Comparing crop rotations between organic and conventional farming

**DOI:** 10.1038/s41598-017-14271-6

**Published:** 2017-10-23

**Authors:** Pietro Barbieri, Sylvain Pellerin, Thomas Nesme

**Affiliations:** 1 INRA, UMR 1391 ISPA, CS 20032, 33882 Villenave d’Ornon, France; 2 Bordeaux Science Agro, Univ. Bordeaux, UMR 1391 ISPA, CS 40201, 33175 Gradignan Cedex, France

## Abstract

Cropland use activities are major drivers of global environmental changes and of farming system resilience. Rotating crops is a critical land-use driver, and a farmers’ key strategy to control environmental stresses and crop performances. Evidence has accumulated that crop rotations have been dramatically simplified over the last 50 years. In contrast, organic farming stands as an alternative production way that promotes crop diversification. However, our understanding of crop rotations is surprisingly limited. In order to understand if organic farming would result in more diversified and multifunctional landscapes, we provide here a novel, systematic comparison of organic-to-conventional crop rotations at the global scale based on a meta-analysis of the scientific literature, paired with an independent analysis of organic-to-conventional land-use. We show that organic farming leads to differences in land-use compared to conventional: overall, crop rotations are 15% longer and result in higher diversity and evener crop species distribution. These changes are driven by a higher abundance of temporary fodders, catch and cover-crops, mostly to the detriment of cereals. We also highlighted differences in organic rotations between Europe and North-America, two leading regions for organic production. This increased complexity of organic crop rotations is likely to enhance ecosystem service provisioning to agroecosystems.

## Introduction

Land-use activities affect a considerable fraction of the global terrestrial surface^1,2^ and are key drivers of habitat and biodiversity loss, water use, global nutrient cycles, greenhouse gas emissions and carbon sequestration^1^. Among all land-use activities, agriculture plays a key role. Because it occupies about 40% of the Earth’s terrestrial surface - the largest single use of land on the planet^1,3^, agriculture contributes to the large appropriation of net primary production by human societies at the global scale^4^. Farming has a tremendous impact on the Earth’s functioning^5–8^ and a large body of literature has shown that current agricultural practices and related land-use activities are dominant forces that are driving the planet beyond its safe operating space^9^.

Cropland-use activities are largely driven by crop rotations^10^. Rotating crops in diverse and complex patterns is one of the oldest agronomic approaches used by farmers to control nutrient and water balances, weed, pest and disease infestations and risk exposure, and to improve system resilience as well as to fulfill human and livestock food and feed needs^11,12^. Because they have a significant impact on agroecosystem functioning as well as on the economic and environmental consequences and performances of cropping systems, diversified rotations are essential to design more sustainable agricultural systems^13^. However, crop rotations have been dramatically simplified over the past 50 years (e.g., through the reduced number of crop species in crop rotations and the increased proportion of land farmed under monoculture)^14,15^ due to the advent of synthetic fertilizers and pesticides^16^ and to the increased disconnection between crop and livestock production^17^. This decrease in the number of crop species in arable rotations has resulted in simplified land-use patterns in modern farming systems, reaching levels that jeopardize the provision of ecosystem services via agroecosystems^18–21^.

Organic farming represents a promising attempt at reconciling food production with environmental protection and multiple ecosystem service delivery^22,23^. Because synthetic fertilizers and pesticides are banned by organic guidelines, rotations are supposed to assume a strategic role in organic production systems. In particular, it is generally supposed that more complex and diversified rotations are adopted in organic systems to sustain crop yields by providing alternative levers for pest control and nutrient management. However, beyond specific local studies, it has never been demonstrated and systematically quantified whether or not crop rotations are more complex in organic farming than in conventional (i.e., non-organic) farming. More generally, because very little systematic data is available about organic rotations, it has never been established to what extent crop rotations and resulting land-use differ between organic and conventional farming. Yet, such knowledge would be critical to assess whether or not organic farming expansion would result in more diversified and multifunctional landscapes than conventional farming. Better understanding of organic crop rotations and land-use composition is also a key – and currently lacking - component to assess the capacity of organic farming to feed the planet^20,24,25^.

Data on crop rotations are scarce, highly dispersed and poorly unified, mostly due to the lack of global datasets. Knowledge gaps are especially large when addressing developing countries and organic systems^26^. Crop rotation data are most commonly collected by farm surveys, experimental plots^27^ and field maps^28^, and are therefore difficult to retrieve at large spatial scales. Remote sensing has been attempted to collect land-use intensity, i.e., cropping frequency and short crop rotation, but only at the regional scale^29–31^. To overcome these difficulties, we developed a global database using a meta-analysis approach by collecting data on the composition of crop rotations (i.e. regardless of the temporal sequence of crops within rotations) from the scientific literature about organic vs. conventional farming performances. Our database is composed of data from 77 publications with information about 238 unique rotations and covering 26 countries worldwide (Supplementary Fig. S1). We supplemented this analysis by constructing a database on organic and conventional global land-use using data from FAOSTAT and FiBL (see Methods section). This second database provided information about organic vs. conventional crop areas for a series of six annual crop categories at the national scale for 50 countries on five continents. Even if the direct comparison of the two datasets has some limitations –because the rotation dataset assesses temporal crop diversity at the field scale, whereas the land-use dataset assesses spatial diversity at the national scale– pairing these two data sources helps to estimate how results from local-scale studies translate into large scale census. By analyzing this rotational database, complemented by the land-use information, we aimed to (i) estimate to what extent rotations differ between organic and conventional farming; (ii) investigate whether such differences vary in different global regions; and (iii) verify whether global land-use data were consistent with the rotation results. This study focuses on temporary arable crops (excluding perennial and permanent crops and fodders) that together provide the bulk of calories and proteins to humans and livestock animals and that cover 70 and 92% of the global cropland area in organic and conventional farming, respectively.

## Results

### Organic rotations are more diversified than their conventional counterparts

**Figure 1:**
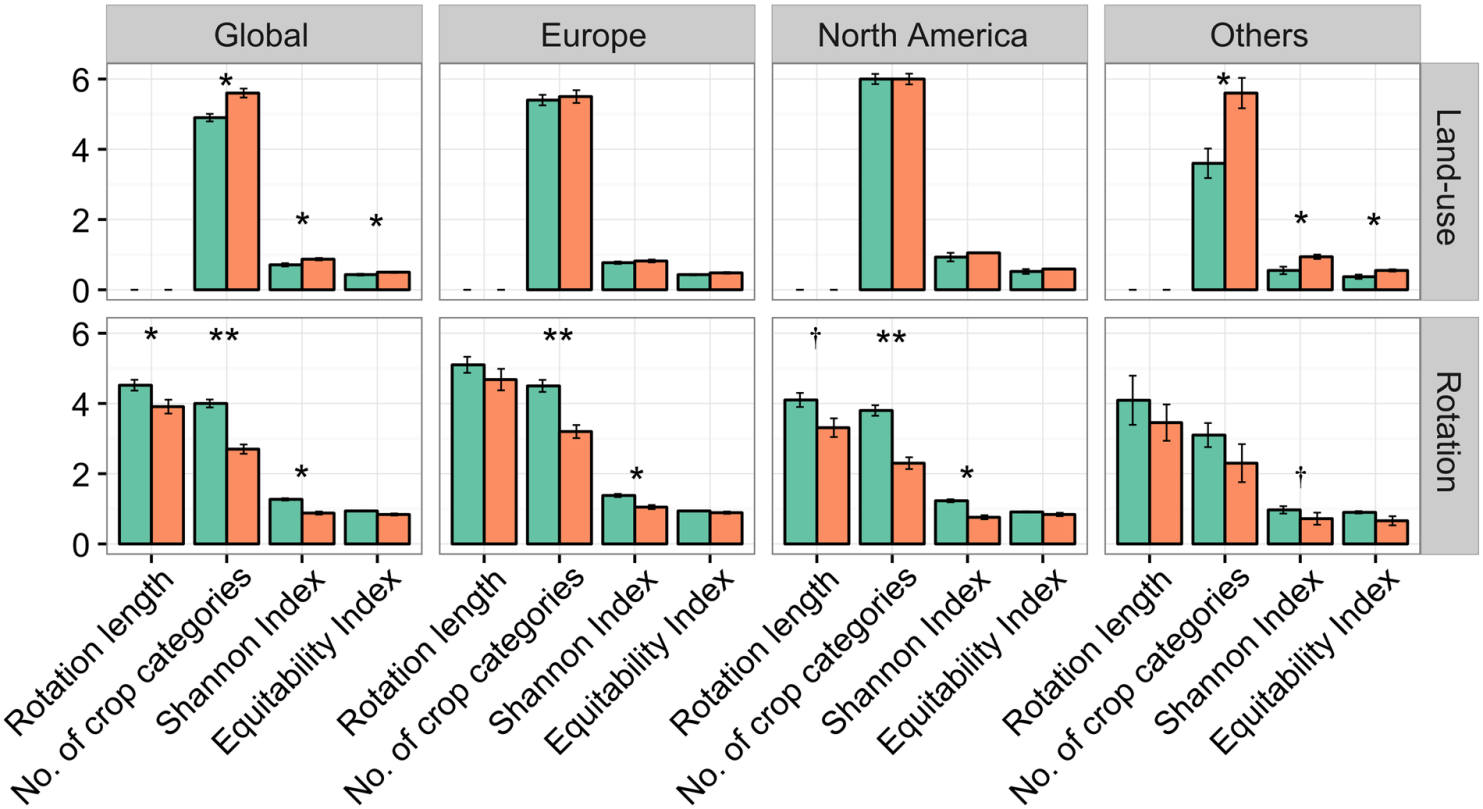
Average ( ± standard error of the mean) rotation length [in years], total number of crop categories in organic (green), and conventional (orange) rotations and land-use, as well as the Shannon Index (H) and the Equitability Index (EH) calculated at the global scale and by global region using the rotation and the land-use datasets. H and EH are calculated based on the timeshare of each crop in the rotation (for the rotation dataset), or based on the relative harvested area of each crop category (for the land-use dataset). The total number of crop categories considered was n = 11 in the rotation dataset and n = 6 in the land-use dataset. **P < 0.01; *P < 0.05; ^†^P < 0.1.

Our results showed that rotations are more diversified in organic than in conventional farming. On average at the global scale, we found that organic rotations last for 4.5 ± 1.7 years, which is 0.7 years or 15% more than their conventional counterparts, and include 48% more crop categories (Fig. 1), thus resulting in higher crop diversity over space, as well as over time (assessed by the Shannon diversity index). This result is in great part due to the higher abundance of catch (defined as any non-harvested cover crop or green manure between two main crops) and undersown cover crops. Our results also showed that organic farming exhibits a more even distribution of the different crop categories (higher Equitability Index in Fig. 1), even if differences between production systems are not significant. In contrast, conventional rotations have a lower diversity, especially in the global region “Others”, i.e., in tropical and subtropical countries. However, the land-use dataset did not confirm the higher diversity of organic systems. In fact, land-use tends to be slightly less diverse in organic systems than in their conventional counterparts, in particular for the global tropical and sub-tropical ‘Others’ region. We found similar results for the equitability of crop categories, although most differences were not significant (Fig. 1). This result might be because the land-use dataset does not contain information on some crop categories, i.e., fodders, catch crops, etc., that contribute to the higher diversity in the rotation dataset. Additionally, especially in the tropics, organic farming is strongly focused on a few export commodities such as vegetables, permanent crops, spices and fruits^32^. Such specialization on a small set of permanent crops might explain the discrepancy between the two datasets when focusing on arable farming systems only.

### Organic and conventional rotations have different crop compositions

We found that the composition of rotations significantly differed between farming systems (Table S1). Organic rotations are composed of primary cereals (i.e. wheat, maize and rice; 29 ± 2% of the rotation length), secondary cereals (i.e. spelt, barley, rye, triticale, oat, sorghum, millet and pseudocereals; 17 ± 2%), pulses (15 ± 2%) and temporary fodders (24 ± 2%), whereas the remaining 15% is shared among oilseeds, root crops, industrial crops and vegetables (Fig. S2). Our results also showed that catch crops and undersown cover crops are 2.4 and 8.7 times more frequent in organic systems compared to conventional systems, respectively, even though their total number in rotations remains low. These rotation characteristics based on our meta-analysis dataset were in good agreement with the land-use data. The latter confirmed that cereals (primary and secondary) compose the greatest fraction of organic cropland use (up to 61 ± 4%) and showed that the share of grain pulses was similar in the two datasets, even though the land-use share of oil crops and vegetables was higher than the rotation dataset (Fig. S2).

### At the global scale, organic rotations have fewer cereals and more temporary fodders

**Figure 2:**
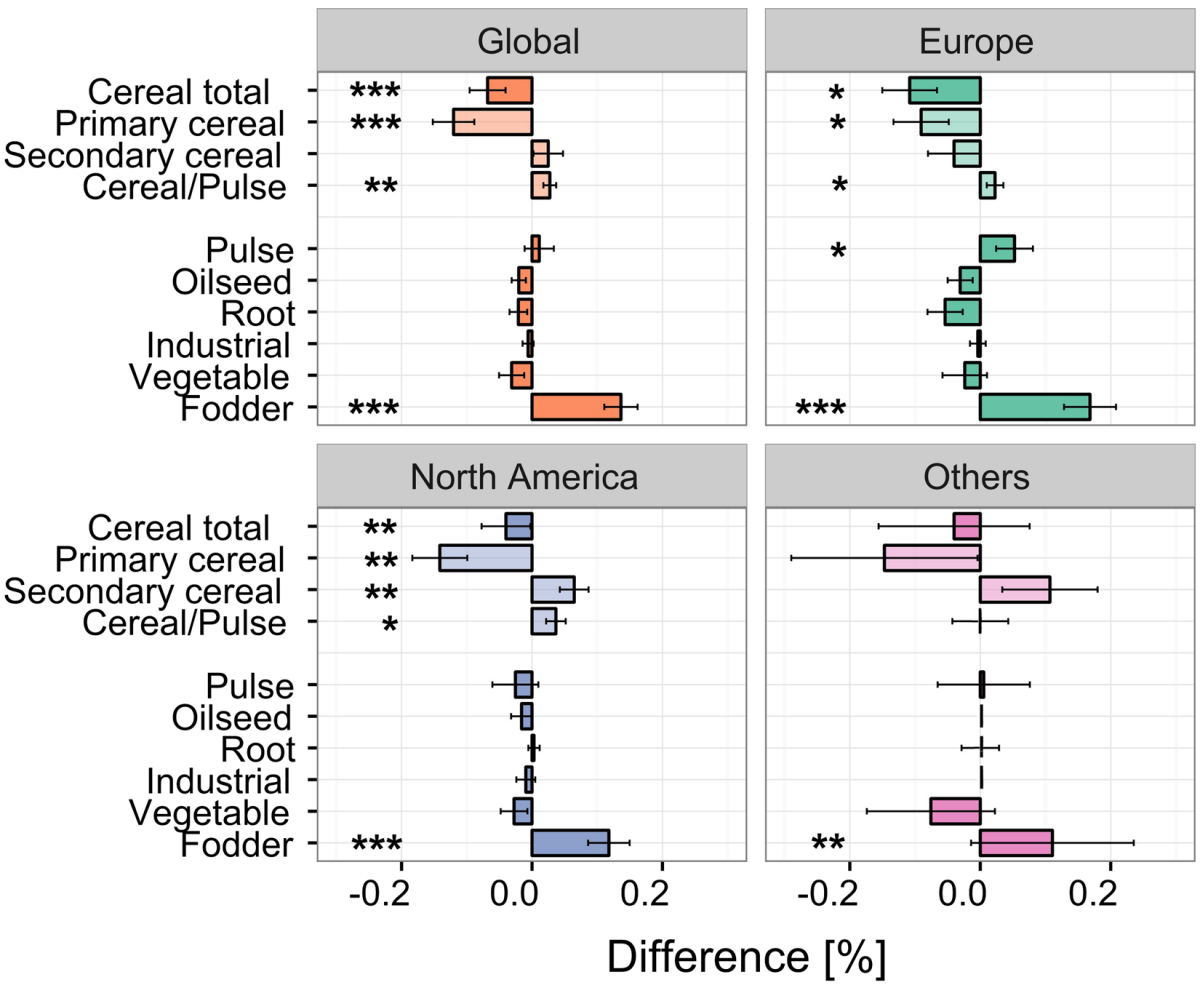
Difference (organic minus conventional, ± standard error of the mean) in crop categories between organic and conventional rotations at the global scale and by global regions (in % of the total rotation length) based on the rotation dataset. The cereal total is the sum of all cereal categories. The shaded sub-categories – ‘Primary cereal’, ‘Secondary cereal’ and ‘Cereal/Pulse’ - refer to primary cereals (wheat, rice, maize), secondary cereals (spelt, barley, rye, triticale, oat, sorghum, millet and pseudocereals), and cereals intercropped with a pulse, respectively. ‘Fodder’ crops refer to temporary fodder crops (such as alfalfa, clover and ryegrass). Number of observations (organic; conventional): Global (127; 111), Europe (53; 46), North America (63; 54), Others (11; 11). ***P < 0.001; **P < 0.01; *P < 0.05.

**Figure 3:**
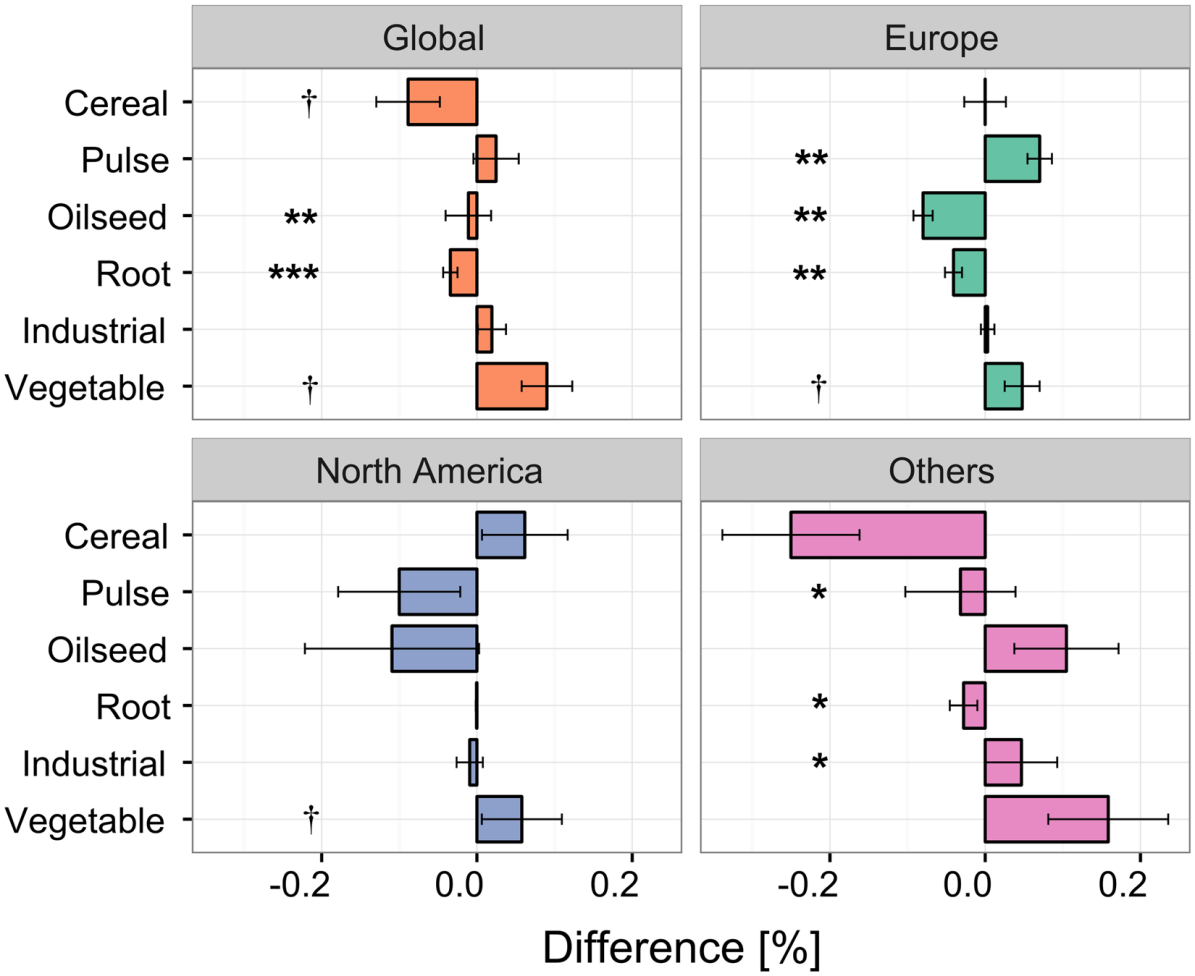
Difference (organic minus conventional, ± standard error of the mean) in crop categories between organic and conventional land-use at the global scale and by global region (in % of harvested area under each crop category in relation to the total cropland area farmed organically or conventionally, respectively) based on the land-use dataset. Number of countries: Global (50), Europe (29), North America (2), Others (19). ***P < 0.001; **P < 0.01; *P < 0.05; ^†^P < 0.1.

Our analysis showed that organic rotations have a 10% lower abundance of cereals compared to their conventional counterparts at the global scale (Fig. 2). This result was due to a marked decrease in primary cereal species, wheat, maize and rice (that were 1.38 times less abundant in organic rotations), although secondary cereals such as barley, rye and oats exhibited a slight increase of 1.19 times in organic rotations (Fig. 2). We also found a higher frequency (4.3 times) of cereal intercropping with legume crops than in conventional systems. In addition, we found that organic rotations have 2.8 times more temporary fodder crops (such as alfalfa, clover, clover-grass, Italian ryegrass, etc.) than conventional systems (Fig. 2), which generally occupy land for an entire year. An important share of organic rotations is also dedicated to catch and undersown cover crops, which are 3.2 and 12.1 times more abundant than in conventional rotations, respectively. These results represent critical information about organic systems since most land-use datasets about croplands critically lack data on temporary fodders and non-harvested crops such as cover or catch crops. We also found that, at the global scale, grain pulses (e.g., soybean, beans and peas) are slightly more abundant in organic rotations although the difference was not statistically significant (Table S1). Finally, we found that organic rotations include slightly less oilseed and root crops (Fig. 2). These results from the meta-analysis of the scientific literature were confirmed by the global land-use data, which showed 16% lower frequency of cereals in organic compared to conventional systems at the global scale (Fig. 3) (although additional details about primary vs. secondary cereals and intercropping were not available in the land-use datasets). The land-use dataset also confirmed that grain pulses are slightly more abundant, while oilseed and root crops are slightly less abundant in organic farming compared to conventional farming (Fig. 3).

### Organic rotations have more nitrogen-fixing crops

**Figure 4:**
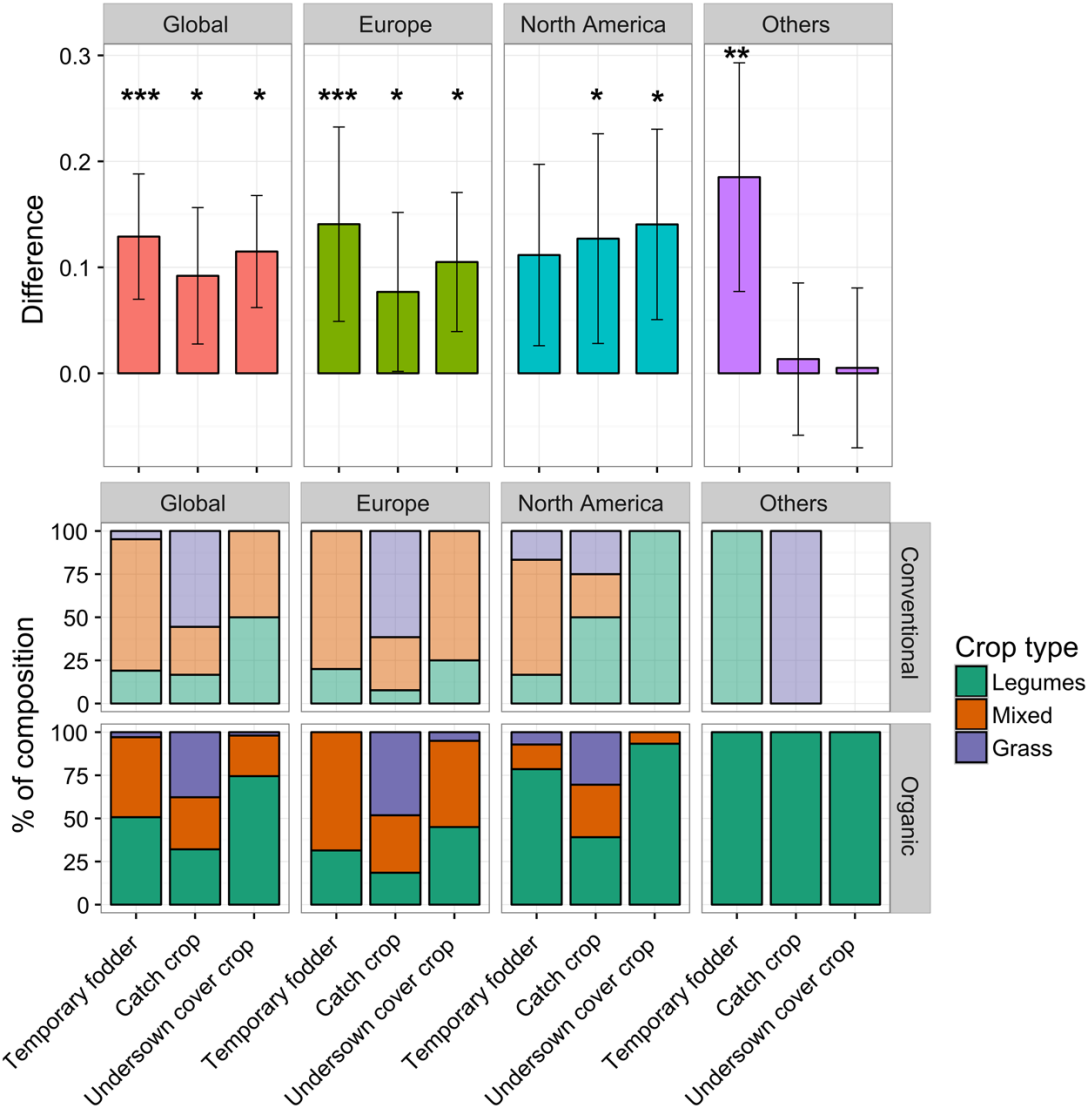
*Above*: Average differences (organic minus conventional, ± standard error of the mean) between the organic and conventional share of fodders, catch and undersown cover crops (in % of the total rotation length) at the global scale and by global region. *Below*: Contribution of grass, mixed (any intercropping of legume and grass) and legume species to temporary fodders, catch crops and undersown cover crop compositions in organic and conventional rotations at the global scale and by global region. Number of observations (organic; conventional): Global (127; 111), Europe (53; 46), North America (63; 54), Others (11; 11). ***P < 0.001; **P < 0.01; *P < 0.05.

Although organic rotations do not significantly exhibit a higher share of grain pulses at the global scale (Fig. 2), our results showed that nitrogen-fixing crops are more abundant in organic farming than in conventional farming. This is due to temporary fodder compositions (Fig. 4) that include more legumes than their conventional counterparts. It is also due to catch and undersown cover crops that are both more frequent and are more often composed of nitrogen-fixing species than in conventional systems (Fig. 4), as well as to the higher frequency of cereal intercropping with legume crops. When combined with a simple estimation of the amount of nitrogen (N) fixed by these leguminous crops, we estimate that, overall, leguminous grain pulses, fodders, catch and undersown cover crops provide 2.6 times more nitrogen to soils farmed organically than they do in conventional rotations. Unfortunately, these crop types have not been tracked in the land-use datasets, making it difficult to assess how representative the results from our meta-analysis are for the crops grown on actual organic vs. conventional farms.

### These differences vary among global regions

Beyond the differences highlighted between organic and conventional farming at the global scale, our study also revealed that these differences strongly vary according to the global regions (Tables S1, S2). For example, we found that cereals were far less abundant in European organic rotations compared to conventional farming, while the difference was much smaller and nuanced in North America (Figs 2, 3). This was due to different behaviors for primary vs. secondary cereals on the two continents: European organic rotations exhibited lower abundance (compared to conventional farming) of both primary and secondary cereals, while secondary cereals were more abundant in North America (Fig. 2). The difference among continents was even more striking regarding pulses: while grain pulses were 65% more abundant in organic rotations and land-use in Europe, we found a 13% lower frequency for these crops in North America. This result is probably due to strong differences in the frequency of these crops in conventional farming – low in Europe, high in North America - largely explained by greater and more stable yield performances of grain pulses in North America and due to difference in both public and economic policies^33^.

## Discussion

Despite their key role in cropping system performances, crop rotations lack systematic analysis in the scientific literature. Our study made it possible to address part of this knowledge gap by comparing organic vs. conventional rotations. In particular, our meta-analysis approach allowed to retrieve systematic information on rotations from a large body of scientific papers and reports. In addition, the comparative approach adopted in this study, which also included an assessment of organic vs. conventional land-use in different crop types at the national scale, was essential to provide information on both organic and conventional production and to highlight system differences between organic and conventional farms. Importantly, our results emphasized the role of temporary fodders, catch and undersown cover crops in organic systems - crops that are typically not included in national land-use databases on organic or conventional agriculture^34,35^. This specific information is of great importance since these non-harvested crops often play critical and multifunctional roles in both organic and conventional farming.

However, our study has some limitations. Firstly, rotation data are difficult to identify based on abstract screening of publications since crop rotations are typically not the focus of a study and information about crop rotations is generally presented in the Materials and Methods section. Some data may therefore have been discarded during our literature search. Secondly, scientific papers mainly report information from experimental field trials, which are not necessarily representative of real farming rotations^36^. In our dataset, 88% of rotations was derived from experimental data, whereas the remaining 12% was derived from on-farm data. Experimental scientific studies today are often focused on crop species that are difficult to manage organically (such as cereals and oilseeds), and cereal-based rotations may therefore be overrepresented. Additionally, the choice of crops within experimental studies may reflect that trials are often carried out in situations where the use of grazing livestock is restricted. Studies addressing a better characterization of real organic farm rotations are clearly necessary. Thirdly, most studies included in our analysis were carried out in North America and Europe, while developing and emergent countries are poorly represented (Fig. S1). Additional studies are particularly required in tropical regions where a large proportion of the organic land area and the majority of organic producers are located^36^. Our parallel analysis based on land-use data made it possible to at least partly address these problems since it allowed to include information on the crop types grown in the countries under-represented in the meta-analysis dataset. However, the comparison of the two datasets is not straightforward. Indeed, while most rotation data were extracted from agronomic papers aiming at comparing cropping systems that were designed based on sound agronomic knowledge and that were possibly designed to test new cropping systems, land-use developed by farmers may be driven by non-agronomic drivers, e.g., economic factors. In addition, the rotation dataset provides temporal data from small-scale studies whereas the land-use dataset brings spatial results about the global crop area. Yet, making the parallel between the two datasets is unique to estimate how local results translate into global, spatial census. Despite all the above-mentioned shortcomings, our analysis represents an important – and to our knowledge, pioneering - step in the characterization of organic farming system land-use patterns.

The deep differences in rotations and land-use that we found between organic and conventional production systems are in line with many organic principles and regulations that often require diverse crop rotations^37^. Our analysis showed that organic systems represent more diversified farming systems with a higher diversity and evenness of crop categories than conventional systems, and with longer rotations. These more diversified systems are associated with multiple benefits^38^. More diverse crop rotations are important management tools for controlling weeds, pests and diseases by creating biotic barriers and interrupting their cycles without the use of synthetic pesticides^38–40^. Additionally, the fact that we found organic rotations to be longer and more diversified than their conventional counterparts indicates that organic systems are likely to be more resilient to abiotic stresses^41^ as well, by especially being more capable of buffering the effect of climate stresses such as increased temperature and rainfall variability^42^. Altogether, these diversification strategies are likely to result in the improved provisioning of ecosystem services to both agroecosystems and the wider environment^21,43^. Specifically, enhanced diversification and the resulting service provisioning may help to narrow the yield gap between organic and conventional farming systems, as suggested by Ponisio *et al*.^44^ who found lower gaps when diversification practices such as intercropping and diversified crop rotations were implemented in organic systems but not in conventional systems. Adopting strategies to narrow the organic-to-conventional yield gap can therefore have the co-benefit of reducing the loss of biodiversity often associated with conventional cropping systems. More diversified agricultural systems could also potentially result in positive impacts on global food security since a higher diversification of food commodities provides more micronutrients than production systems with less diversity^45^. Indeed, this higher diversification might also be due to how organic crop rotation might have been affected by the legislative development of organic farming, especially trough public subsidies to certain areas and crop types.

The differences in rotations and land-use that we found between organic and conventional production systems show that organic systems have been designed to satisfy the fertilization requirements determined by the different organic principles and regulations. Indeed, meeting crop nutrient demand, in particular for nitrogen, by appropriate and ‘organic-compatible’ practices is a key lever to close the organic-to-conventional yield gap^44,46^. The greater abundance of nitrogen-fixing crop species found in organic rotations reflects the multifunctional role played by temporary fodders to achieve organic principles, not only to control pests but to fix N in soils as well^47^. In particular, the fact that we very frequently observed the use of legume and mixed legume-grass fodders in organic systems means that cropping practices have been designed to compensate for the lower external supply of N to crops due to the prohibition of synthetic N fertilizers under organic management. Our analysis also showed that this greater use of leguminous fodders is accompanied by a lower frequency of grain pulses found in organic rotations. Such a choice is agronomically sound because temporary fodders provide additional services besides N fertilization (weed control, disease break crop, carbon sequestration in soils, feed production, etc.)^47^ and because the occurrence of several pulse crops in a short timespan can favor problematic diseases such as anthracnose and downy mildew^48^. Additionally, organic farms are often mixed farms (especially in Europe), and the greater use of fodders is also in line with the need to produce animal feed within the region, as required, for example, by European organic regulations^49^. Finally, the greater use of catch and undersown cover crops found in organic systems suggests that farmers have adopted agronomic strategies to limit N leaching– a problem due to difficulties in synchronizing fertilization practices and crop nutrient uptake^50,51^ - and soil erosion, and to compensate for the high economic cost of external organic N sources.

Finally, this analysis of organic rotation and land-use analysis, although limited by the availability of data at the global scale, represents a necessary step to conduct organic vs. conventional comparisons at the cropping system rather than at the crop level^52,53^. This step is important because estimating the crop production capacity of organic agriculture requires consideration of whole production systems and not just individual crop species^53^. A better understanding of organic crop rotations is also important to estimate the crop nutrient requirements and ecosystem service provisioning that would result from the expansion of organic farming. The differences in crop rotations under organic management that we observed in our study would result in drastic modifications of crop nutrient requirements and services provided by agricultural landscapes, as well as in possible imbalances in human vs. animal needs due to the strong differences in the crop categories produced. However, these changes have been poorly captured so far in prospective studies that assess food security in organic production scenarios at large scales. Such changes are indeed more complex than a simple increase in N-fixing crops, a parameter that is supposed to encompass all land-use changes when modeling conversion to organic agriculture up until now^24,54^. More detailed information about temporary fodders at the global scale and by global region is necessary to better assess food and feed provisioning over the entire organic cropping system^46,52,53^. This is because longer rotations that include more fodder crops might undermine food provisioning by competing with grain crop species on the one hand, and have strong consequences for the livestock sector on the other hand. By alleviating these caveats, our results provide a foundation to build more realistic hypotheses about land-use change and to improve future models to assess the contribution of organic farming to feed the planet.

In summary, to our knowledge, this study represents the first comparative analysis of organic vs. conventional rotations at the global scale. The results of our analysis clearly revealed that the ban of synthetic inputs in organic production forced organic rotations to adopt major changes compared to their conventional counterparts: increased rotation length, higher crop diversity, more frequent temporary fodders, nitrogen-fixing crops and intercropping. The increased complexity and diversity of crop rotations that result from the conversion to organic farming is likely to provide strong environmental benefits and enhanced ecosystem services. Such information is of key importance to guide the conversion to organic farming as a way to achieve global food security without compromising the protection of the environment.

## Materials and Methods

### Rotation dataset

#### Literature search and publication screening

We collected the data on organic vs. conventional rotations through both an original literature search and the reuse of existing databases on similar topics. The original literature search was undertaken using the ‘Web of Science’ portal. We used a complex Boolean search containing (i) the term *ecological*, *biological* or *organic* next to (ii) the term *farming*, *agriculture*, *cropping* or *production*, in combination with (iii) the term *rotation*, *comparison* or *conventional*. The last search was conducted on October 28, 2016, turning up 431 papers. In addition to this literature search, we retrieved the databases referenced by Seufert *et al*.^46^, De Ponti *et al*.^52^, and Ponisio *et al*.^44^ about organic vs. conventional crop yields. These databases accounted for an additional 264 publications, leading to a total of 695 papers.

The abstracts of these 695 initially retrieved papers were first screened to verify whether crop rotation data were actually present, resulting in the selection of 301 records. These 301 papers were further screened by checking if (i) they provided different organic and conventional treatments, i.e. if equal rotation were reported, the study was discarded, (ii) they reported complete rotation schemes, and (iii) the organic treatment was either certified organic or in line with the definition of organic agriculture given in the *Basic Standards for Organic Production and Processing* of the International Federation of Organic Agricultural Movement (IFOAM)^55^. Papers’ methods that provide equal rotations in both conventional and organic cropping systems may -in most cases- be interpreted as a choice to attenuate the difference between the two farming systems, since they might focus on different parameters but the rotation itself. We also excluded multiple publications reporting on the same trials to avoid double counting. Publications reporting rotations in multiple countries were considered as different entries, using the country as the discriminating criterion. As suggested by De Ponti *et al*.^52^, data prior to 1985 were not included because they were considered outdated, with the exception of long-term trials. Following such criteria, the screening yielded only 77 publications for further analysis, including 238 unique rotations covering 26 countries worldwide (Fig. S3). The majority of data came from Europe (42%) and North America (49%). The complete list of studies is provided in the Supplementary Table S3.

#### Data extraction

Information on rotation length, number of crops, catch and undersown cover crops were recorded from each publication, regardless of their temporal sequence in the rotation. We defined as *crop* any crop species that stands on a field over a cropping season, with a duration of maximum one year. Therefore, if several crop species were grown simultaneously on the same field in the same year, only the main crop was considered (with the exception of cereals intercropped with pulses and temporary fodders that were recorded as such). We also recorded information on non-harvested crops. To derive the total number of crop species present in each rotation (proxy for crop species diversity), we counted only the net number of crops (e.g., if one crop species was present for two or more years in the rotation, it was counted as just one). We also counted the real number of crops to estimate the timeshare of each crop category in the rotation. For instance, if one crop species was present for two years in the rotation, we counted it as one to derive the total number of crop species in the rotation (proxy for crop species diversity), but we counted it as 2 in order to calculate the timeshare of such crop in the rotation. We defined as *undersown cover crop* any relay intercropped species, and as *catch crop* any green manure or winter catch crop. Crops were then classified according to the following crop categories: (i) primary cereals (wheat, rice, maize); (ii) secondary cereals (spelt, barley, rye, triticale, oat, sorghum, millet and pseudocereals); (iii) intercropped cereals with pulses; (iv) pulses (including soybeans); (v) oilseeds; (vi) root crops (potato, sugar beets, cassava, sweet potato); (vii) industrial crops (flax, tobacco); and (viii) temporary fodders. For temporary fodders, catch crops and undersown cover crops, we recorded whether the corresponding species was a legume, a grass or a mixture of the two (e.g., clover-grass mixture). For each rotation, the time share of each crop category was calculated by dividing the number of crops in each crop category by the total rotation length. Finally, the location of each study was retrieved through the country in which the study took place. Countries were grouped according to three main global regions: Europe, North America and Others (Fig. S1). Countries other than European and North American were grouped into one single region due to the low number of data retrieved in such countries (n = 22, 9% of the dataset), in order to obtain balanced data groups for the statistical analysis. Overall, the number of organic rotations was slightly higher than the conventional one (53% and 47%, respectively). This is because some studies reported one conventional rotation compared to two, or more, organic rotations.

We estimated the nitrogen fixed by pulses, temporary fodders, catch and undersown cover crops by assigning a leguminous species to each crop category (i.e., pea for pulses, alfalfa for fodders and vetch for catch and cover crops) and using the model of Høgh-Jensen^56^. Calculations were computed considering a field size of 1 ha.

#### Land-use dataset

We created an original database on organic vs. conventional land-use by collecting country-level statistical data from the Research Institute of Organic Agriculture (FiBL, Switzerland)^34^ for organic agricultural land-use and from FAOSTAT^35^ for conventional agricultural land-use, for the years 2010–2014. Since the original structure of the two databases differed, datasets were restructured in order to allow data comparability of arable crop categories. To do so, land-use data, i.e., the harvested area for each crop category, were expressed according to the following crop categories: cereals (primary and secondary), pulses (including soybeans), oilseeds, root crops, industrial crops and vegetables. No information on organic temporary fodders was available in either of the databases. Hence, we could not compare the two systems’ land-use based on this specific crop category. Information at the crop species level in the FiBL database was not detailed enough to run an analysis at that level.

The data about land-use under conventional agriculture were retrieved by subtracting the area under organic farming (provided by FiBL) from the data on arable land-use provided by FAOSTAT for each country. The across-years land-use average was calculated and used for further analysis. For each country and production system (organic and conventional), the land-use share of each crop category was calculated as the area under the specific crop category divided by the cropland area under the total number of crop categories considered. The data were filtered by removing countries for which the share of organic area was lower than 0.5% of the total agricultural area. Overall, land-use from 50 countries were compared. European and North America countries represent 62% of the dataset, followed by Asian (16%), Latin American (10%), African (10%) and Oceanian (2%) countries. Countries were grouped according to the same three global regions defined for the rotation dataset (i.e., Europe, North America and Others) to facilitate comparisons of datasets as much as possible. Nevertheless, the region “Others” was not directly comparable between the two datasets since the composition of the countries was slightly different.

### Statistical analysis

We examined richness and diversity of organic and conventional rotations and land-use by using Shannon’s diversity and equitability indices. Shannon’s diversity index (H, Eq. 1) helped to assess the relative abundance of crop categories, providing an indication about species diversity, while the Equitability index (E_H_, Eq. 2) helped to assess whether the different crop categories have an even share in both rotations and land-use. The two indices were calculated as follows:

1 $$H=\,-\sum _{i=1}^{s}{p}_{i}\,\mathrm{ln}({p}_{i})$$

where p_i_ represents the proportion of crop category *i*

2 $${E}_{H}=\frac{H}{{H}_{max}}=\frac{H}{\mathrm{ln}(S)}$$

where S is the total number of crop categories.The data expressed as counts (i.e., rotation length, total number of crops and number of catch and undersown cover crops) were analyzed using a Generalized Mixed Model following a Poisson distribution. The production system (organic vs. conventional), global region and their interaction were included as fixed factors. The ‘study’ was included as a random effect to account for possible “study effects” and data overdispersion.

The data expressed as percentages (i.e., share of the different crop categories in each rotation and land-use) were analyzed using a Permutational Analysis of Variance (non-parametric MANOVA) with distance matrices to test the null hypothesis of no difference between production systems, global regions and their interactions. This made it possible to partition distance matrices among sources of variation and to fit a linear model to the different matrices. The partial R-squared (r^2^) obtained indicates the percentage of variance that is explained by the factors. The significance of each explanatory variable was computed from F-tests based on sequential sums of squares from permutations of the raw data^57^. The analysis was run using the Bray-Curtis dissimilarity index, and the number of permutations to compute the significance tests was set to 999. We tested the differences in the share of each crop category between production systems, global regions and their interactions using a non-parametric Kruskal-Wallis test, followed by a post-hock pairwise Dunn test.

Differences between production systems in terms of Shannon diversity were tested by using a Linear Mixed Model (production system as the fixed factor; studies’ number as a random effect to account for possible “study effects”), and a Linear Model (production system as the fixed factor), respectively, for the rotation and the land-use datasets, followed by a classical analysis of variance. Normality of data was verified through a Shapiro-Wilk test and residual check plots. The equitability indices were far from being normally distributed and their differences between organic vs. conventional systems were therefore tested using a non-parametric Kruskal-Wallis test. We calculate the Shannon and the equitability indices using both all the data across the 4-year period and the across-year average. Since we did not find any effect due to the variation over time, we finally kept the calculation done using the across-year average.

All the analyses were performed in R Open 3.3.2 (MRAN 2016), using the “lme4” package for mixed models^58^, the “rcompanion” package for non-parametric models^59^, the “FSA” package to evaluate the significance of the effects^60^, and the “vegan” package for descriptive community ecology^61^.

### Data availability

The authors declare that the main data supporting the findings of this study are available within the article and its Supplementary Information files. Extra data are available from the corresponding author upon request.

## Electronic supplementary material

Supplementary Tables and Figures

Crop Rotation dataset
