# Supplementary material for: Comparing crop rotations between organic and conventional farming
Source: Sci Rep. 2017 Oct 23;7:13761. doi: 10.1038/s41598-017-14271-6 (PMC5653822; doi:10.1038/s41598-017-14271-6)
Supplement: Supplementary file 1 — Supplementary Tables and Figures [file 41598_2017_14271_MOESM1_ESM.doc]

Supplementary Information

Comparing crop rotations between organic and conventional farming

**Pietro Barbieri^a*^, Sylvain Pellerin^a^, Thomas Nesme^b^**

^a^ INRA, UMR 1391 ISPA, CS 20032, 33882 Villenave d’Ornon, France

^b^ Bordeaux Science Agro, UMR 1391 ISPA, CS 40201, 33175 Gradignan Cedex, France

* Corresponding author: Pietro Barbieri; Email: [pietro.barbieri@inra.fr](mailto:pietro.barbieri@inra.fr); Tel.: +33 (0)5 57 12 26 55; ORCID ID: orcid.org/0000-0003-3248-4487

**Table S1**: Rotation summary data and results of the non-parametric ANOVA (Kruskal-Wallis) showing the effect of the system (organic vs. conventional farming), region (Europe, North America vs. Others) and system × region interactions, and their significance levels on the percentage of the timeshare under each crop category (rotation dataset).

| **Model** |  |  | **Rotation length** | **Number of crop categories** | **Primary cereal** | **Secondary cereal** | **Cereal/Pulse** | **Pulse** | **Oilseed** | **Root** | **Industrial** | **Vegetable** | **Fodder** | **Number of catch crops** | **Number of undersown cover crops** |
| --- | --- | --- | --- | --- | --- | --- | --- | --- | --- | --- | --- | --- | --- | --- | --- |
|  | *Organic rotation* |  | 4.5 | 3.5 | 32% | 15% | 4% | 16% | 2% | 5% | 1% | 4% | 21% | 0.78 | 0.61 |
|  | *Conventional rotation* |  | 3.8 | 2.9 | 44% | 13% | 1% | 15% | 4% | 7% | 2% | 7% | 7% | 0.32 | 0.07 |
|  |  |  |  |  |  |  |  |  |  |  |  |  |  |  |  |
|  | *Fixed effects* | *DF* | *ChiSq* | *ChiSq* |  |  |  |  |  |  |  |  |  | *ChiSq* | *ChiSq* |
| Poisson GLM | System | 1 | 3.6 * | 6.3 * |  |  |  |  |  |  |  |  |  | 15.6 *** | 15.9 *** |
|  | Region | 2 | 9.0 * | 11.6 ** |  |  |  |  |  |  |  |  |  | 13.6 ** | 12.6 ** |
|  | System × Region | 2 | 0.6 | 1.6 |  |  |  |  |  |  |  |  |  | 6.87 * | 7.5 * |
|  |  |  |  |  |  |  |  |  |  |  |  |  |  |  |  |
|  | *Group* | *DF* |  |  | *ChiSq* | *ChiSq* | *ChiSq* | *ChiSq* | *ChiSq* | *ChiSq* | *ChiSq* | *ChiSq* | *ChiSq* |  |  |
| Kruskal-Wallis | System | 1 |  |  | 12.5 *** | 3.7 | 8.5 ** | 0.6 | 2.2 | 1.6 | 0.1 | 1.6 | 31.4 *** |  |  |
|  | Region | 2 |  |  | 41.2 *** | 39.3 *** | 39.3 *** | 24.6 *** | 3.9 | 51.1 *** | 5.4 † | 9.33 ** | 8.5 * |  |  |
|  | Interaction (S × R) | 5 |  |  | 54.6 *** | 46.1 *** | 46.2 *** | 26.7 *** | 6.6 | 55.0 *** | 6.1 | 10.9 † | 42.3 *** |  |  |

DF degrees of freedom; *** P < 0.001; ** P < 0.01; * P < 0.05; † P < 0.1

**Table S2**: Land-use summary data and results of the non-parametric ANOVA (Kruskal-Wallis) showing the effect of the system (organic vs. conventional farming), region (Europe, North America vs. Others) and system × region interactions, and their significance levels on the percentage of the area under each crop category (land-use dataset).

| **Model** |  |  | **Cereal** | **Pulse** | **Oilseed** | **Root** | **Industrial** | **Vegetable** | |
| --- | --- | --- | --- | --- | --- | --- | --- | --- | --- |
|  | *Organic land use* |  | 61% | 11% | 9% | 2% | 3% | 14% | |
|  | *Conventional land use* |  | 69% | 8% | 10% | 6% | 1% | 5% | |
|  |  |  |  |  |  |  |  |  | |
|  | *Group* | *DF* | *ChiSq* | *ChiSq* | *ChiSq* | *ChiSq* | *ChiSq* | *ChiSq* | |
| Kruskal-Wallis | System | 1 | 0.43 † | 0.01 | 7.79 ** | 21.2 *** | 0.53 | 3.80 † | |
|  | Region | 4 | 13.2 ** | 6.33 * | 5.10 † | 3.48 | 6.85 * | 5.52 † |  |
|  | Interaction (S × R) | 9 | 16.9 ** | 36.5 *** | 20.3 ** | 26.1 *** | 12.1 * | 10.9 † |  |

DF degrees of freedom; *** P < 0.001; ** P < 0.01; * P < 0.05; † P < 0.10;

**Table S3: Results of the permutational analysis of variance (ADONIS) on the rotation and on the land-use datasets showing the significance of the effects of system (organic vs. conventional farming), region (Europe, North America vs. Others) and of the interaction system × region, and their share of explained variance (R^2^).**

|  | **Rotation dataset** | | | | **Land-use dataset** | | | |
| --- | --- | --- | --- | --- | --- | --- | --- | --- |
| *Effect* | *DF* | *Sum of squares* | *P-value* | *R^2^* | *DF* | *Sum of squares* | *P-value* | *R^2^* |
| System | 1 | 2.42 | 0.001 *** | 0.051 | 1 | 0.38 | 0.001 *** | 0.051 |
| Region | 2 | 5.51 | 0.001 *** | 0.114 | 2 | 0.85 | 0.002 ** | 0.090 |
| System × Region | 2 | 0.31 | 0.487 | 0.006 | 2 | 0.61 | 0.007 ** | 0.065 |

DF degrees of freedom; *** P < 0.001; ** P < 0.01; * P < 0.05; † P < 0.10;

**Table S4**: List of studies included in the rotation database (author, year, journal and title) and the country in which the studies were conducted.

| **Study** | **Author** | **Year** | | **Journal** | **Title** | **Country** |
| --- | --- | --- | --- | --- | --- | --- |
| 1 | Acs et al. | | 2007 | Biological Agriculture & Horticulture | Comparison of conventional and organic arable farming systems in the Netherlands by means of bio-economic modeling | Netherlands |
| 2 | Andrist-Rangel et al. | | 2007 | Agriculture, Ecosystems & Environment | Long-term K dynamics in organic and conventional mixed cropping systems as related to management and soil properties | Sweden |
| 3 | Benoit et al. | | 2015 | Agriculture, Ecosystems & Environment | Nitrous oxide emissions and nitrate leaching in an organic and a conventional cropping system (Seine basin, France) | France |
| 4 | Chirinda et al. | | 2010 | Agriculture, Ecosystems & Environment | Soil properties, crop production and greenhouse gas emissions from organic and inorganic fertilizer-based arable cropping systems | Denmark |
| 5 | Garnier et al. | | 2016 | Environmental Science & Policy | Reconnecting crop and cattle farming to reduce nitrogen losses to river water of an intensive agricultural catchment (Seine basin, France): past, present and future | France |
| 6 | Küstermann et al. | | 2008 | Renewable Agriculture and Food Systems | Modeling carbon cycles and estimation of greenhouse gas emissions from organic and conventional farming systems | Germany |
| 7 | Lazzerini et al. | | 2014 | Italian Journal of Agronomy | A simplified method for the assessment of carbon balance in agriculture: An application in organic and conventional micro-agroecosystems in a long-term experiment in Tuscany, Italy | Italy |
| 8 | Lee et al. | | 2014 | The Journal of Horticultural Science and Biotechnology | Effects of hairy vetch, rye, and alternating cultivation of rye-vetch cover crops on soil nutrient concentrations and the production of red pepper (Capsicum annuum L.) | South Korea |
| 9 | Mancinelli et al. | | 2010 | Applied Soil Ecology | Soil carbon dioxide emission and carbon content as affected by conventional and organic cropping systems in Mediterranean environment | Italy |
| 10 | Osler et al. | | 2008 | Applied Soil Ecology | Soil micro arthropod assemblages under different arable crop rotations in Alberta, Canada | Canada |
| 11 | Pardo et al. | | 2014 | Outlook on Agriculture | Economic profitability analysis of rainfed organic farming in SW Spain | Spain |
| 12 | Smith et al. | | 2004 | Renewable Agriculture and Food Systems | Profitability and risk of organic production systems in the northern Great Plains | Canada |
| 13 | Wortman et al. | | 2012 | Renewable Agriculture and Food Systems | Soil fertility and crop yields in long-term organic and conventional cropping systems in Eastern Nebraska | USA |
| 14 | Zentner et al. | | 2011 | Renewable Agriculture and Food Systems | Effects of input management and crop diversity on economic returns and riskiness of cropping systems in the semi-arid Canadian Prairie | Sweden |
| 15 | Acher et al. | | 2007 | Agronomy Journal | Leaching and crop uptake of N, P and K from organic and conventional cropping systems on a clay soil | Germany |
| 16 | Aronsson et al. | | 2007 | American Society of Agronomy | Soil Use and Management | Sweden |
| 17 | Auerswald et al. | | 2006 | Soil and Tillage Research | Influence of cropping system on harvest erosion under potato | USA |
| 18 | Baeckström et al. | | 2006 | Communications in Soil Science and Plant Analysis | Nitrogen Use Efficiency in an 11-Year Study of Conventional and Organic Wheat Cultivation | Denmark |
| 19 | Cavigelli et al. | | 2009 | Renewable Agriculture and Food Systems | Long-term economic performance of organic and conventional field crops in the mid-Atlantic region | USA |
| 20 | Chirinda *et al.* | | 2008 | 16th IFOAM Organic World Congress | Effects of organic matter input on soil microbial properties and crop yields in conventional and organic cropping systems | France |
| 21 | Clark et al. | | 1999 | Agriculture, Ecosystems and Environment | Nitrogen, weeds and water as yield-limiting factors in conventional, low-input, and organic tomato systems | Australia |
| 22 | Delmotte et al. | | 2011 | European Journal of Agronomy | On farm assessment of rice yield variability and productivity gaps between organic and conventional cropping systems under Mediterranean climate | Denmark |
| 23 | Deria et al. | | 2014 | Organic Wheat Production and Soil Nutrient Status in a Mediterranean Climatic Zone | | Norway |
| 24 | Doltra | | 2010 | ICROFS News | A better nitrogen use to improve organic wheat production | Canada |
| 25 | Eltun et al. | | 2002 | Agriculture, Ecosystems and Environment | A comparison of environmental, soil fertility, yield, and economical effects in six cropping systems based on an 8-year experiment in Norway | Norway |
| 26 | Entz et al. | | 2005 | Proceedings of the First Scientific Conference of the International Society of Organic Agriculture Research | Influence of organic management with different crop rotations on selected productivity parameters in a long-term Canadian field study | USA |
| 27 | Fjelkner-Modig et al. | | 2000 | Acta Agriculturae Scandinavica, Section B - Soil & Plant Science | The Influence of Organic and Integrated Production on Nutritional, Sensory and Agricultural Aspects of Vegetable Raw Materials for Food Production | Switzerland |
| 28 | Gelfand et al. | | 2010 | Environmental Science and Technology | Energy efficiency of conventional, organic, and alternative cropping systems for food and fuel at a site in the U.S. Midwest | Sweden |
| 29 | Kirchmann | | 2007 | Agronomy Journal | Comparison of Long-Term Organic and Conventional Crop–Livestock Systems on a Previously Nutrient-Depleted Soil in Sweden | Switzerland |
| 30 | Kitchen et al. | | 2003 | Australian Journal of Agricultural Research | Comparing wheat grown in South Australian organic and conventional farming systems. 1. Growth and grain yield | Italy |
| 31 | Mazzoncini | | 2006 | Aspects of Applied Biology 79, What will organic farming deliver? COR 2006 | Sunflower under conventional and organic farming systems: results from a long term experiment in Central Italy | New Zeeland |
| 32 | Murphy | | 2007 | Field Crops Research | Evidence of varietal adaptation to organic farming systems | USA |
| 33 | Nguyen | | 1995 | Agriculture, Ecosystems and Environment | Energy and labour efficiency for three pairs of conventional and alternative mixed cropping (pasture-arable) farms in Canterbury, New Zealand | USA |
| 34 | Peck et al. | | 2006 | HortScience | Apple Orchard Productivity and Food Quality under Organic, Conventional, and Integrated Management | USA |
| 35 | Pimentel | | 2005 | BioScience | Environmental, Energetic, and Economic Comparisons of Organic and Conventional Farming Systems | USA |
| 36 | Porter et al. | | 2003 | Agronomy Journal | Organic and Other Management Strategies with Two- and Four-Year Crop Rotations in Minnesota | Australia |
| 37 | Posner et al. | | 2008 | Agronomy Journal | Organic and conventional production systems in the Wisconsin integrated cropping systems trials: I. Productivity 1990-2002 | Turkey |
| 38 | Ryan et al. | | 2004 | Journal of the Science of Food and Agriculture | Grain mineral concentrations and yield of wheat grown under organic and conventional management | USA |
| 39 | Sermenli et al. | | 2007 | Journal of Sustainable Agriculture | Effect of Strip intercropping and organic farming systems on quantity and quality of maize yield in a Mediterranean region of Turkey | USA |
| 40 | Smith et Gross | | 2006 | Weed Science | Weed community and corn yield variability in diverse management systems | USA |
| 41 | Smolik et al. | | 1995 | American Journal of Alternative Agriculture | The relative sustainability of alternative, conventional, and reduced-till farming systems | Estonia |
| 42 | Tamm et al. | | 2009 | Agronomy Research | Spring cereals performance in organic and conventional cultivation | Denmark |
| 43 | Teasdale | | 2007 | Agronomy Journal | Potential long-term benefits of no-tillage and organic cropping systems for grain production and soil improvement | USA |
| 44 | Thorup-Kristensen | | 1999 | An organic crop rotation aimed at self-sufficiency in nitrogen | Organic eprints | Greece |
| 45 | Treadwell et al. | | 2008 | HortScience | Nutrient management with cover crops and compost affects development and yield in organically managed sweet potato systems | Canada |
| 46 | Welsh et al. | | 2009 | Agronomy Journal | High yielding organic crop management decreases plant-available but not recalcitrant soil phosphorus | Spain |
| 47 | Delate et Cambardella | | 2004 | Agronomy Journal | Agroecosystem performance during transition to certified organic grain production | Czech Republic |
| 48 | Korsaeth et al. | | 2012 | Applied and Environmental Soil Science | N, P, and K budgets and changes in selected topsoil nutrients over 10 years in a long-term experiment with conventional and organic crop rotations | Slovakia |
| 49 | Martini et al. | | 2004 | Field Crops Research | Yield increases during the organic transition: Improving soil quality or increasing experience? | USA |
| 50 | Posner et al. | | 2005 | WICST 10th Technical Report (http://wicst.wisc.edu/wp-content/uploads/wicst-yields-yield-variability-and-yield-trends-1990-20021.pdf) | The Wisconsin Integrated Cropping Systems Trials: yields, yield variability, and yield trends 1990-2002 | Denmark |
| 51 | Thorup-Kristensen et al. | | 2012 | European Journal of Agronomy | Crop yield, root growth, and nutrient dynamics in a conventional and three organic cropping systems with different levels of external inputs and N re-cycling through fertility building crops | Italy |
| 52 | Torstensson | | 2006 | Agronomy Journal | Nutrient use efficiencies and leaching of organic and conventional cropping systems in Sweden | USA |
| 53 | Campanelli et al.. | | 2012 | Journal of Sustainable Agriculture | Crop Production and Environmental Effects in Conventional and Organic Vegetable Farming Systems: The Case of a Long-Term Experiment in Mediterranean Conditions (Central Italy) | USA |
| 54 | Coulter et al. | | 2011 | Agronomy Journal | Agronomic performance of cropping systems with contrasting crop rotations and external inputs | India |
| 55 | Drinkwater et al. | | 2000 | Plant and Soil | Effects of tillage intensity on nitrogen dynamics and productivity in legume-based grain systems | USA |
| 56 | Liebhardt et al. | | 1989 | Agronomy Journal | Crop production during conversion from conventional to low-input methods | USA |
| 57 | Lotter et al. | | 2003 | American Journal of Alternative Agriculture | The performance of organic and conventional cropping systems in an extreme climate year | USA |
| 58 | Mahoney et al. | | 2004 | Renewable Agriculture and Food Systems | Profitability of organic cropping systems in southwestern Minnesota | China |
| 59 | Reganold et al. | | 1987 | Nature | Long-term effects of organic and convention farming on soil erosion | USA |
| 60 | Temple et al. | | 1994 | American Journal of Alternative Agriculture | An interdisciplinary, experiment station-based participatory comparison of alternative crop management systems for California's Sacramento valley | USA |
| 61 | Gallaher et al. | | 2015 | Renewable Agriculture and Food Systems | Organic management and legume presence maintained phosphorus bioavailability in a 17-year field crop experiment | Japan |
| 62 | Knudsen et al. | | 2014 | Journal of Cleaner Production | Carbon footprints of crops from organic and conventional arable crop rotations - Using a life cycle assessment approach | Estonia |
| 63 | Moreno et al. | | 2011 | Soil and Tillage Research | Rainfed crop energy balance of different farming systems and crop rotations in a semi-arid environment: Results of a long-term trial | Norway |
| 64 | Sánchez de Cima et al. | | 2015 | International Agrophysics | Organic farming and cover crops as an alternative to mineral fertilizers to improve soil physical properties | Idia |
| 65 | Eltun and Nordheim | | 1999 | Designing and testing crop rotations for organic farming Danish Research Centre for Organic Farming DARCOF Report no. 1 | Yield results during the first eight years crop rotation of the Apelsvoll cropping system experiment | USA |
| 66 | Melero et al. | | 2006 | Soil and Tillage Research | Chemical and biochemical properties in a silty loam soil under conventional and organic management | France |
| 67 | Stalenga | | 2007 | Journal of Plant Nutrition | Applicability of different indices to evaluate nutrient status of winter wheat in the organic system | USA |
| 68 | Benoit et al. | | 2016 | Agricultural Systems | A participative network of organic and conventional crop farms in the Seine Basin (France) for evaluating nitrate leaching and yield performance | USA |
| 69 | Cooper et al. | | 2011 | Journal of Agricultural and Food Chemistry | Effect of Organic and Conventional Crop Rotation, Fertilization, and Crop Protection Practices on Metal Contents in Wheat (Triticum aestivum) | USA |
| 70 | Jaradat et al. | | 2011 | Agronomy Journal | Statistical modeling of yield and variance instability in conventional and organic cropping systems | Poland |
| 71 | Wander et al. | | 1994 | Soil Science Society of America Journal | Organic and Conventional Management Effects on Biologically Active Soil Organic Matter Pools | Estonia |
| 72 | Wu et al. | | 2003 | Geoderma | Soil management effects on the non-limiting water range | USA |
| 73 | Ryan et al. | | 2009 | Weed Research | Weed-crop competition relationships differ between organic and conventional cropping systems | Kenya |
| 74 | Wortman | | 2010 | Renewable Agriculture and Food Systems | Increased weed diversity, density and above-ground biomass in long-term organic crop rotations | Norway |
| 75 | Adamtey et al. | | 2016 | [Agriculture, Ecosystems & Environment](http://www.sciencedirect.com/science/journal/01678809) | Productivity, profitability and partial nutrient balance in maize-based conventional and organic farming systems in Kenya | Norway |
| 76 | Korsaeth et al. | | 2008 | Relations between nitrogen leaching and food productivity in organic and conventional cropping systems in a long-term field study | Agriculture, Ecosystems and Environment | Norway |
| 77 | Lien et al. | | 2006 | Comparison of risk in organic, integrated and conventional cropping systems in eastern Norway | Journal of Farm Management | Norway |


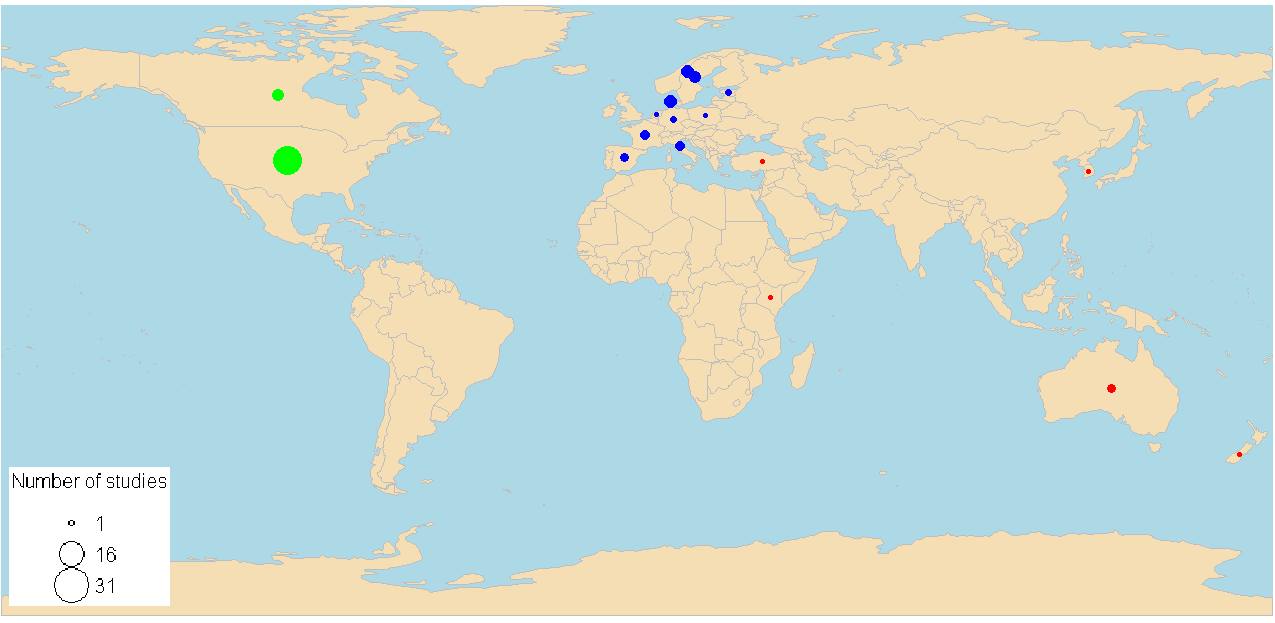


**Figure S1.** Map showing the 77 study sites that were included in the rotation dataset. The map was generated using R: A Language and Environment for Statistical Computing 3.3.2 (R Core Team, Vienna, Austria, 2016, https://www.R-project.org) and the “rworldmap” package (South A. Package ‘rworldmap. *CRAN Repos.*, 2016).


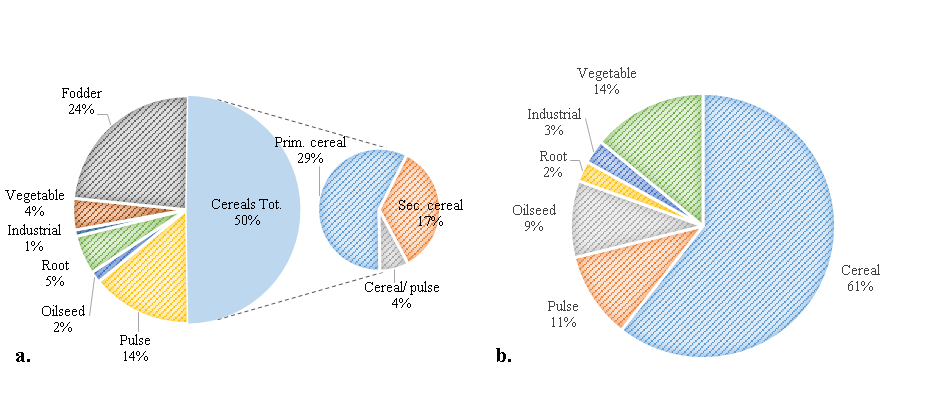


Figure S2. Average composition of organic (a) rotations and (b) land use by crop category. Shares are calculated as the percentage of total crop rotation length occupied by each crop category and the share of the area occupied by each crop category, respectively.

**
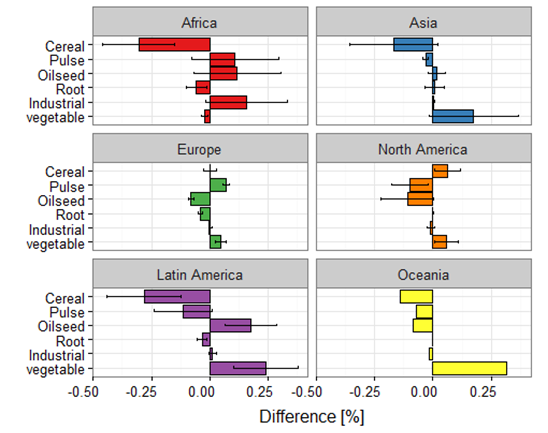
**

Figure S3. Difference (organic minus conventional, ±standard error of the mean) in crop categories between organic and conventional land use for the extended global regions (in % of harvested area of each crop in relation to the total cropland area under organic vs. conventional farming) based on the land-use dataset. Number of countries: Africa (7), Asia (6), Europe (29), North America (2), Latin America (9), Oceania (2).
